# Beyond cardiovascular risk: is ‘normal’ blood pressure too high for reproductive success?

**DOI:** 10.1093/hropen/hoag057

**Published:** 2026-06-17

**Authors:** Berthold Hocher, Shujuan Ma

**Affiliations:** Clinical Research Center for Reproduction and Genetics in Hunan Province, Reproductive and Genetic Hospital of CITIC-Xiangya, Changsha, China; Key Laboratory of Reproductive and Stem Cell Engineering, Central South University, Changsha, China; University Medical Centre Mannheim of the University of Heidelberg, Mannheim, Germany; Institute of Medical Diagnostics, Berlin-Potsdam, Germany; Clinical Research Center for Reproduction and Genetics in Hunan Province, Reproductive and Genetic Hospital of CITIC-Xiangya, Changsha, China; Key Laboratory of Reproductive and Stem Cell Engineering, Central South University, Changsha, China

**Keywords:** blood pressure, fertility, live birth, miscarriage, preconception care, IVF, vascular health

## Abstract

The relationship between blood pressure (BP) and reproductive health is increasingly recognized as a clinically relevant and biologically plausible axis influencing fertility outcomes. Evidence from ART cohorts suggests that reproductive success may decline across a continuum of increasing BP, extending below conventional hypertensive thresholds. Observational studies have linked male prehypertension with impaired semen quality and reduced ART success, and maternal prepregnancy systolic BP with lower live birth rates, higher miscarriage risk, and a greater burden of hypertensive pregnancy complications. These findings raise a provocative question: are BP levels considered acceptable for long-term cardiovascular prevention also optimal for implantation, placentation, and live birth? This Opinion article argues that preconception BP should be considered a potentially informative reproductive vascular marker, while clearly distinguishing BP as a possible causal determinant from BP as a surrogate of underlying vascular-metabolic health. Potential mechanisms include endothelial dysfunction, impaired nitric oxide signalling, reduced uterine perfusion, vascular inflammation, and altered remodelling affecting both the maternal environment and gamete quality. At the same time, causality remains unproven because current evidence is predominantly observational and vulnerable to residual confounding. A focused research agenda is therefore needed to test whether preconception BP optimization can improve reproductive outcomes and to determine whether reproductive medicine requires endpoint-specific BP thresholds.

## Introduction

Hypertensive disorders of pregnancy remain a major cause of maternal and perinatal morbidity and mortality, but the reproductive relevance of blood pressure (BP) may begin long before pregnancy complications become clinically manifest. Across preconception and fertility-treatment settings, emerging data suggests that BP is not merely a vital background sign; it may be a marker of reproductive fitness and a modifiable determinant of treatment success.

IVF provides a useful framework for studying this relationship because distinct stages of human reproduction can be observed separately, from gamete quality and fertilization to embryo development, implantation, miscarriage, and live birth. Within this setting, elevated prepregnancy systolic BP has been associated with reduced oocyte maturation, lower live birth rates, and a higher burden of hypertensive pregnancy complications. Evidence from naturally conceiving populations points in the same direction.

This article advances a debate-oriented and hypothesis-generating thesis: BP thresholds developed for cardiovascular prevention may not necessarily be the thresholds most informative for reproductive outcomes. The argument is not that high-normal BP has already been proven to cause reduced fertility or that reproductive medicine should immediately redefine treatment thresholds. Rather, we propose that preconception BP may be a clinically accessible marker of reproductive vascular health, a possible causal contributor in selected patients, and a useful exposure variable for future causal studies. In this framework, reproductive outcomes such as implantation, miscarriage, hypertensive pregnancy disorders, and live birth are considered potential vascular endpoints that deserve systematic prospective and interventional evaluation before any endpoint-specific BP thresholds are proposed for clinical use.

## Evidence for a reproductive blood pressure continuum

Large ART cohorts have consistently shown an inverse association between prepregnancy systolic BP and live birth. Importantly, this relationship does not appear to begin only once overt hypertension is present. Data from IVF populations indicate that even women in the high-normal range may have lower live birth rates and higher miscarriage risk than women with lower BP levels. Recent large cohorts further suggest a dose–response relationship across the BP spectrum. ART cohorts also provide important information on less frequent but clinically relevant outcomes, including ectopic pregnancy (Perkins *et al*., 2015).

**Figure 1.**
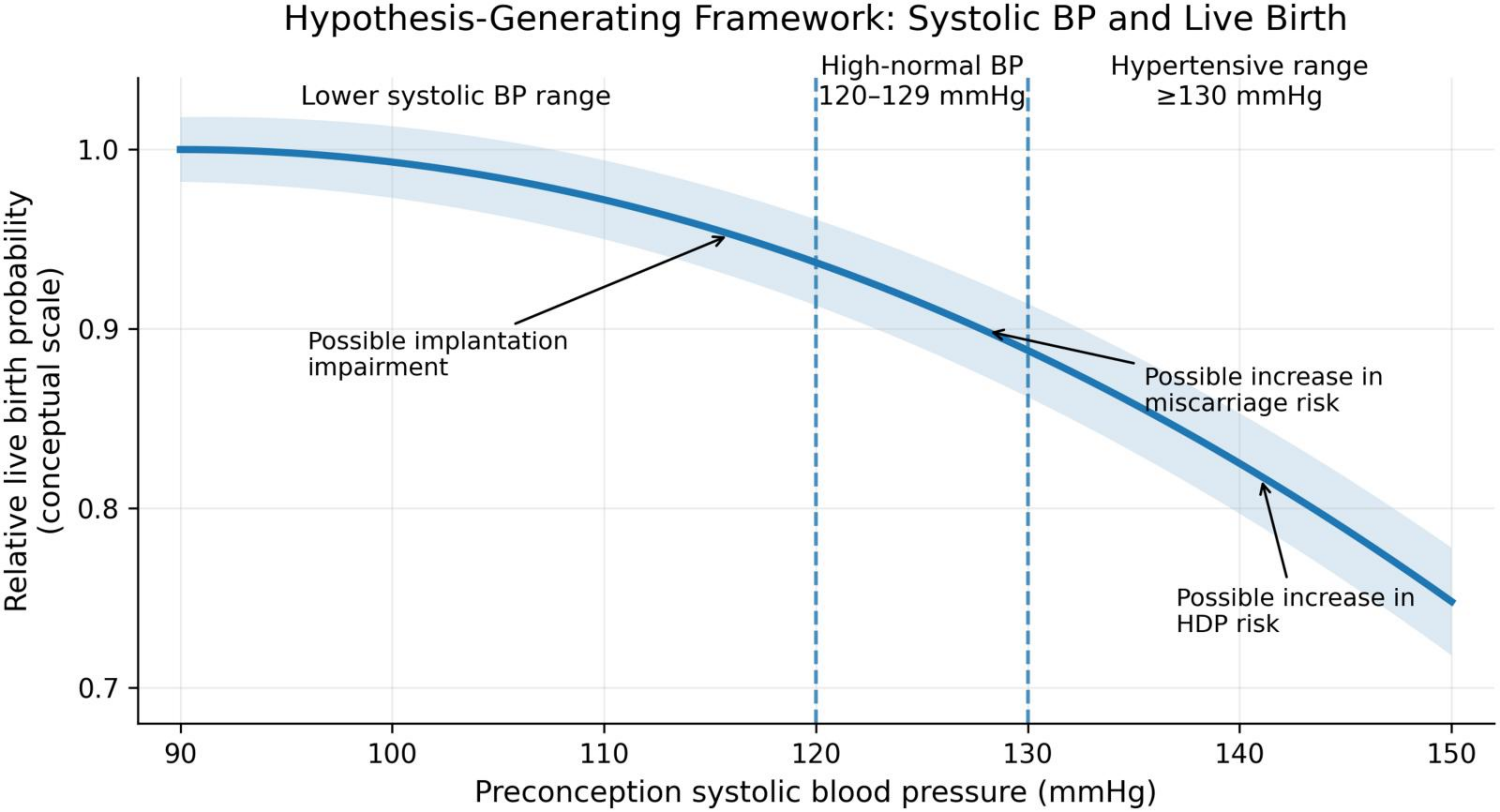
**Hypothesis-generating framework linking preconception systolic blood pressure and reproductive outcome.** The figure illustrates the hypothesis that live birth probability may decline across a continuum of increasing preconception systolic blood pressure, including ranges traditionally classified as normotensive or high-normal for cardiovascular risk assessment. The curve and shaded area are schematic only and do not represent a fitted dose–response model, a specific dataset, statistical confidence intervals, or validated clinical thresholds. Vertical dashed lines indicate conventional systolic blood pressure categories for orientation, not proposed reproductive treatment cut-offs. The annotations indicate possible reproductive correlates of increasing blood pressure, including impaired implantation, miscarriage risk, and hypertensive pregnancy disorder risk. The figure is intended to guide future research and should not be interpreted as evidence that blood pressure reduction improves live birth outcomes. BP, blood pressure; HDP, hypertensive disorders of pregnancy.

Evidence from naturally conceiving populations provides external support for this concept. In the EAGeR trial, higher preconception BP, particularly diastolic BP and mean arterial pressure, was associated with increased pregnancy loss among women attempting conception (Nobles *et al*., 2018). Other preconception and early-pregnancy BP studies have linked higher BP to time to pregnancy, hypertensive pregnancy disorders, and adverse pregnancy outcomes (Hong *et al*., 2019; Greenberg *et al*., 2021). Collectively, these studies support the hypothesis of a reproductive cardiovascular continuum, in which even modest BP elevation may reflect vascular dysfunction relevant to fertility and early pregnancy maintenance (see also Table 1 and Figure 1). However, this continuum should be interpreted as a conceptual framework for research, not as evidence that BP itself is always causal or that new clinical treatment thresholds are already justified.

**Table 1.** Summary of studies linking blood pressure with reproductive outcomes.

Study	Population	Age	BMI	Key findings
Ma *et al.* (2024)	73 462 women undergoing IVF/ICSI	20–45 years; median/IQR NR in accessible text	Normotensive group 21.7 ± 2.51 kg/m²; hypertensive group 22.9 ± 2.67 kg/m²; anti-hypertensive treatment group 23.1 ± 2.74 kg/m²	Continuous inverse association between prepregnancy systolic BP and live birth across the BP spectrum.
Chen *et al.* (2022)	2418 women undergoing fresh embryo transfer	Live birth 29.1 ± 3.40 years; no-live birth 29.6 ± 3.84 years	Live birth 21.4 ± 2.4 kg/m²; no-live birth 21.5 ± 2.39 kg/m²	High-normal BP associated with reduced live birth compared with lower BP.
Fang *et al.* (2026)	>40 000 women in an IVF cohort	32.3 ± 4.7 years overall; 32.2 ± 4.5 to 33.7 ± 5.1 years across BP categories	23.3 ± 3.3 to 27.1 ± 4.2 kg/m² across BP categories	Higher BP associated with lower live birth and higher pregnancy loss risk.
Nobles *et al.* (2018)	1228 women attempting natural conception in EAGeR	28.7 ± 4.8 years	26.3 ± 6.5 kg/m²	Higher preconception DBP and MAP associated with increased pregnancy loss risk.
Wang *et al.* (2025)	Male partners undergoing first single blastocyst FET cycles	Control 33.1 ± 4.4 years; prehypertension 33.5 ± 4.6 years	Control 25.0 ± 3.3 kg/m²; prehypertension value to verify	Male prehypertension associated with impaired semen parameters and reduced ART success.

BP, blood pressure; SBP, systolic blood pressure; DBP, diastolic blood pressure; MAP, mean arterial pressure; FET, frozen embryo transfer; NR, not reported. Age and BMI are shown as mean ± SD, median (IQR), or category-specific values according to the format used in the original publication.

## Blood pressure as causal determinant or surrogate marker? Evidence for and against causality

A crucial distinction is whether BP directly contributes to impaired reproductive outcome or whether it mainly marks an upstream vascular-metabolic phenotype. A causal interpretation is biologically plausible because higher BP may be accompanied by increased vascular tone, reduced endothelial-dependent vasodilation, impaired uterine or endometrial perfusion, and less favourable placental vascular adaptation. In this scenario, BP reduction or vascular optimization before conception could theoretically improve implantation, pregnancy maintenance, or hypertensive pregnancy outcomes.

The alternative interpretation is equally important: BP may be a surrogate marker rather than the primary driver. Adiposity, insulin resistance, renal function, vascular ageing, inflammation, endothelial dysfunction, sleep disturbance, and broader cardiometabolic health may simultaneously raise BP and reduce reproductive success. Under this model, lowering BP alone would not necessarily improve live birth unless the shared upstream phenotype is also modified. The available observational studies cannot reliably distinguish these possibilities, even when they adjust for measured confounders.

Several observations support, but do not prove, a causal interpretation. The association has been reported in different settings, including ART cohorts and a prospective natural-conception cohort; BP is measured before the reproductive endpoint, which establishes temporality; some studies describe a graded relationship across BP categories or continuous BP values; and the association is biologically plausible through endothelial dysfunction, impaired nitric oxide (NO) signalling, altered uterine perfusion, and abnormal placental vascular adaptation. The IVF setting further strengthens the research opportunity because oocyte retrieval, embryo development, transfer, implantation, pregnancy loss, and live birth can be observed as distinct steps.

At the same time, important observations argue against overinterpreting the data as causal. Effect sizes are generally modest, associations are not always consistent across systolic-, diastolic-, and mean-BP, or specific reproductive endpoints, and some associations attenuate after adjustment for BMI and other covariates. Residual confounding remains likely because adiposity, insulin resistance, renal function, inflammation, endothelial dysfunction, sleep disturbance, socioeconomic factors, and medication use are incompletely measured in most cohorts. Many studies also rely on one or few clinic BP measurements and cannot fully account for white-coat hypertension, masked hypertension, long-term BP patterns, or vascular health independent of BP. Most importantly, no trial has yet shown that lowering preconception BP improves implantation, reduces miscarriage, or increases live birth.

For this reason, the proposed reproductive BP framework should be understood as a research and risk-phenotyping framework rather than an immediate therapeutic threshold framework. Its purpose is to encourage standardized BP measurement, deeper cardiometabolic and vascular phenotyping, and causal study designs that can determine whether BP is a modifiable cause, a marker of shared risk, or both. Only if prospective and randomized evidence shows that BP optimization improves clinically meaningful reproductive outcomes should reproductive medicine consider endpoint-specific BP thresholds.

## Why this is biologically plausible

The biological plausibility of a BP–fertility axis is supported by vascular mechanisms that are directly relevant to implantation and placentation. A particularly plausible pathway is endothelial dysfunction with reduced NO bioavailability. Normal implantation requires coordinated endometrial receptivity, decidual vascular adaptation, and early trophoblast invasion. These processes depend on adequate microvascular perfusion and endothelial-dependent vasodilation. NO is a central mediator of vascular relaxation and pregnancy-related vascular adaptation; reduced NO bioavailability may increase vascular tone, impair uterine and endometrial perfusion, and promote a more inflammatory and pro-thrombotic vascular environment (Sladek *et al*., 1997; Reynolds and Redmer, 2001; Brown and Garovic, 2014; Vorosc *et al*., 2025). In this context, elevated preconception BP may be interpreted either as a potential contributor to impaired reproductive vascular adaptation or as a marker of an underlying endothelial phenotype that is less favourable for implantation and early placental development.

This pathway also provides a mechanistic bridge between preconception vascular health and later hypertensive pregnancy disorders. Defective early placentation and impaired maternal vascular adaptation are central features in the pathophysiology of preeclampsia and related hypertensive pregnancy complications (Sladek *et al*., 1997; Brown and Garovic, 2014; Magee and von Dadelszen, 2018). Thus, BP measured before fertility treatment may capture part of the same vascular biology that later becomes clinically apparent as implantation failure, miscarriage, or hypertensive pregnancy disease. Importantly, this interpretation remains hypothesis-generating: current clinical evidence does not prove that BP reduction before conception improves live birth, nor does it establish a specific reproductive BP threshold.

Vascular dysfunction may also influence ovarian function and spermatogenesis. Ovarian follicular development and corpus luteum function require finely regulated angiogenesis and perfusion, while spermatogenesis depends on testicular microvascular integrity and endocrine-metabolic homeostasis. These mechanisms may help explain why both maternal prepregnancy BP and male prehypertension have been associated with reproductive outcomes in observational studies (Reynolds and Redmer, 2001; Ma *et al*., 2024; Wang *et al*., 2025).

IVF and embryo selection technologies make this question unusually tractable. By allowing stage-specific observation of oocyte competence, embryo development, implantation, and live birth, ART provides a setting in which vascular effects can be studied more precisely than in unselected natural-conception cohorts. The use of PGT-A can partially disentangle embryonic competence from maternal physiology, strengthening the inference that BP-associated effects may be maternal and vascular rather than exclusively embryonic (Fragouli *et al*., 2020).

## Practical implications for ART care and future research

The current evidence should not be interpreted as a mandate to redefine clinical BP thresholds for reproductive medicine. In particular, it does not yet justify pharmacological BP reduction solely to improve live birth rates, nor should ART be delayed only because a woman has high-normal BP in the absence of other clinical indications. No randomized trial has yet shown that preconception BP lowering improves implantation, reduces miscarriage, or increases live birth.

Nevertheless, the available evidence is sufficient to change how BP is viewed in reproductive care. Preconception BP should be measured carefully and documented consistently rather than treated as a routine vital sign of limited relevance. Overt hypertension should be recognized and managed according to existing cardiovascular and pregnancy-safety guidelines before conception or ART. High-normal BP should not be treated as a disease state, but it may be considered a signal of broader vascular or metabolic risk.

In practical terms, BP optimization in the ART setting would most realistically begin with low-risk lifestyle measures rather than immediate pharmacological treatment. These may include weight optimization where appropriate, dietary counselling including salt reduction and a Dietary Approaches to Stop Hypertension (DASH)-like dietary pattern, regular physical activity, smoking cessation, moderation of alcohol intake, sleep optimization, and assessment of coexisting metabolic or renal risk factors. Such measures may improve general preconception health and long-term cardiovascular risk, but they also require time, motivation, and additional clinical visits. In older women or women with diminished ovarian reserve, delaying ART for prolonged lifestyle intervention could itself reduce reproductive chances. Lifestyle-based BP optimization should therefore be individualized and should avoid creating unnecessary treatment delays, anxiety, or blame for patients.

Pharmacological BP optimization raises additional complexities. Established hypertension should be treated according to current guidelines using pregnancy-compatible medication when conception is planned. However, treating high-normal BP or stage 1 hypertension solely to improve ART outcomes remains experimental. Potential benefits could include improved vascular function, lower risk of hypertensive pregnancy disorders, and possibly improved implantation or pregnancy maintenance. Potential risks include medication adverse effects, inappropriate exposure around conception, overtreatment, hypotension, additional monitoring burden, cost, and delay of embryo transfer. Pharmacological treatment for reproductive benefit should therefore not be introduced into routine ART care until well-powered randomized trials demonstrate that it improves clinically meaningful outcomes, especially live birth, without unacceptable harm or delay.

The research implication is direct: future ART studies should treat BP as a structured preconception exposure rather than as background clinical information. Prospective cohorts should use repeated standardized clinic, home, or ambulatory BP measurements and should collect BMI, insulin resistance, renal function, inflammatory and vascular biomarkers, stimulation protocols, embryo quality, embryo-transfer strategy including fresh versus frozen embryo transfer, implantation, miscarriage, hypertensive pregnancy disorders, other perinatal complications, patient burden, and live birth (Bosdou *et al*., 2020; Yan *et al*., 2026). Within-woman analyses across repeated ART cycles, sibling or partner-based designs, and Mendelian randomization may help separate causal effects from shared confounding. Ultimately, pragmatic randomized trials are needed to test whether structured lifestyle-based, and where appropriate pregnancy-compatible pharmacological, BP optimization improves reproductive outcomes without causing treatment delay, medication-related harm, or excessive patient burden.

Until such evidence is available, the appropriate conclusion is neither to lower treatment thresholds nor to dismiss BP as irrelevant. Clinicians should measure BP carefully, treat established hypertension according to current guidelines, and view high-normal BP as a possible marker of vascular-metabolic health that may justify broader preconception risk optimization. The key unresolved question for the field is whether BP is a modifiable causal determinant of impaired reproductive success, a surrogate marker of the vascular and metabolic phenotype that accompanies reduced fertility, or both.

## Data Availability

No new datasets were generated or analysed for this manuscript. This article is based on published literature.
